# False-negative diagnosis of high anion gap in patients with end-stage kidney disease

**DOI:** 10.1038/s41598-021-84087-y

**Published:** 2021-02-25

**Authors:** You Komatsuzaki, Masato Ikeda, Akihiro Shimizu, Nanae Matsuo, Yukio Maruyama, Takashi Yokoo, Hiroyuki Yamamoto, Nobuhiko Joki, Ryoichi Ando, Daijo Inaguma, Toshihiko Yamaka, Masaaki Nakayama, Fumihiko Koiwa, Shinya Kawamoto, Shigeo Negi, Takashi Shigematsu

**Affiliations:** 1 Division of Nephrology and Hypertension, The Jikei University School of Medicine Kashiwa Hospital, 163-1, Kashiwashita, Kashiwa, Chiba 277-8567 Japan; 2 Division of Nephrology and Hypertension, Department of Internal Medicine, The Jikei University School of Medicine, Tokyo, Japan; 3 Department of Healthcare Quality Assessment, Graduate School of Medicine, The University of Tokyo, Tokyo, Japan; 4 Division of Nephrology, Toho University Ohashi Medical Center, Tokyo, Japan; 5 Department of Nephrology, Musashino Red Cross Hospital, Tokyo, Japan; 6 Department of Internal Medicine, Fujita Health University Bantane Hospital, Nagoya, Japan; 7 Division of Clinical Engineering, Department of Technology, Kanagawa Institute of Technology, Kanagawa, Japan; 8 Department of Nephrology, Division of Internal Medicine, St. Luke’s International University, Tokyo, Japan; 9 Division of Nephrology, Division of Internal Medicine, Showa University Fujigaoka Hospital, Yokohama, Japan; 10 Department of Nephrology, Dokkyo Medical University Koshigaya Hospital, Saitama, Japan; 11 Rinku General Medical Center, Osaka, Japan; 12 Division of Nephrology and Blood Purification Medicine, Wakayama Medical University, Wakayama, Japan

**Keywords:** Acid, base, fluid, electrolyte disorders, Chronic kidney disease, Renal replacement therapy, Physiology, Diseases, Medical research, Nephrology, Risk factors

## Abstract

The traditional anion gap (AG) equation is widely used, but its misdiagnosis in end-stage kidney disease (ESKD) patients has not been investigated fully. Diagnostic accuracy to detect high AG was cross-sectionally evaluated using 3 AG equations in 1733 ESKD patients with an eGFR less than 15 mL/min/1.73 m^2^. The prevalence of high AG was 67.9%, 92.1% and 97.4% by the traditional, albumin-adjusted AG (aAG) and full AG equations, respectively. The sensitivity, specificity, accuracy and Kappa coefficient obtained with the traditional AG vs aAG equation were 0.70 vs 0.94, 0.98 vs 0.93, 0.7 vs 0.94, and 0.103 vs 0.44, respectively. Next, we created a subcohort comprising only patients with high full AG and investigated how the traditional AG equation leads to misdiagnoses. Multivariable-adjusted regression analysis in 1688 patients revealed that independent factors associated with a false-negative AG diagnosis were ARB use, eGFR, blood leukocyte count, serum chloride, bicarbonate, ionized calcium, potassium, albumin and phosphate. 93.2% of our subcohort prescribed any of RAAS inhibitors, Loop diuretics or Alkali which could increase either serum chloride or bicarbonate. Frequent use of these possible AG-reducing medications may conceal high AG state in patients with ESKD unless they have incidental inflammation which may increase AG value.

## Introduction

Calculation of the serum anion gap (AG) remains an inexpensive and effective tool that aids detection of various acid–base disorders, hematologic malignancies, and intoxications^1^. The serum AG represents the unmeasured anions present in plasma and increases as disease advances in chronic kidney disease (CKD), with a decrease in the tubular excretion of anion molecules involving uremic toxins^1,2^ such as indoxyl sulfate (IS), 3-carboxy-4-methyl-5-propyl-2-furanpropionate (CMPF), indoleacetate (IA) and hippurate (HA), members of the uremic toxin family^3,4^.

Finally, unmeasured anion molecules are stored in blood plasma in end-stage kidney disease (ESKD). Thus, non-dialysis ESKD has been a well-known high AG disease and may be an ideal disease to study AG.

However, we sometimes experience patients with a non-high range of traditional AG, even in ESKD, and either the full AG or albumin-adjusted equation could be used to screen for a high AG state in these patients. These patients receive a false-negative misdiagnosis by the traditional AG equation. We hypothesized that the traditional AG equation often diagnose AG state false-negatively in patients with ESKD. Though the traditional AG calculation is popularly used worldwide due to its simple and easy equation, its diagnostic accuracy in patients with ESKD has not been fully evaluated to date. These clinical experiences encouraged us to evaluate both the accuracy of the traditional AG equation and the possible cause for misdiagnosis.

The Japanese Study Group for Assessing Initiation of Renal Replacement Therapy (JSTART) is a clinical research group in which 9 institutions gather to study ESKD^5,6^. We updated the JSTART database by adding the full AG value, and we included 2964 Japanese patients between January 1, 2009 and September 30, 2017, in this study.

The full AG and aAG equations are more accurate than the traditional AG equation, and their association with either mortality or the progression of CKD has been reported^7–10^. We measured these 3 AG equations, examined the prevalence of high AG in non-dialysis patients with ESKD who should have developed high AG, examined the rates of false-negative diagnoses for high anion gaps and evaluated the accuracy of traditional AG by comparing it to full and aAG. Finally, we investigated how the traditional AG equation misdiagnoses the AG state in ESKD. This information will be useful in daily medical practice in various clinical fields.

## Results

### Diversity of the albumin-adjusted and full AG values against the traditional AG value in non-dialysis patients with ESKD

**Figure 1:**
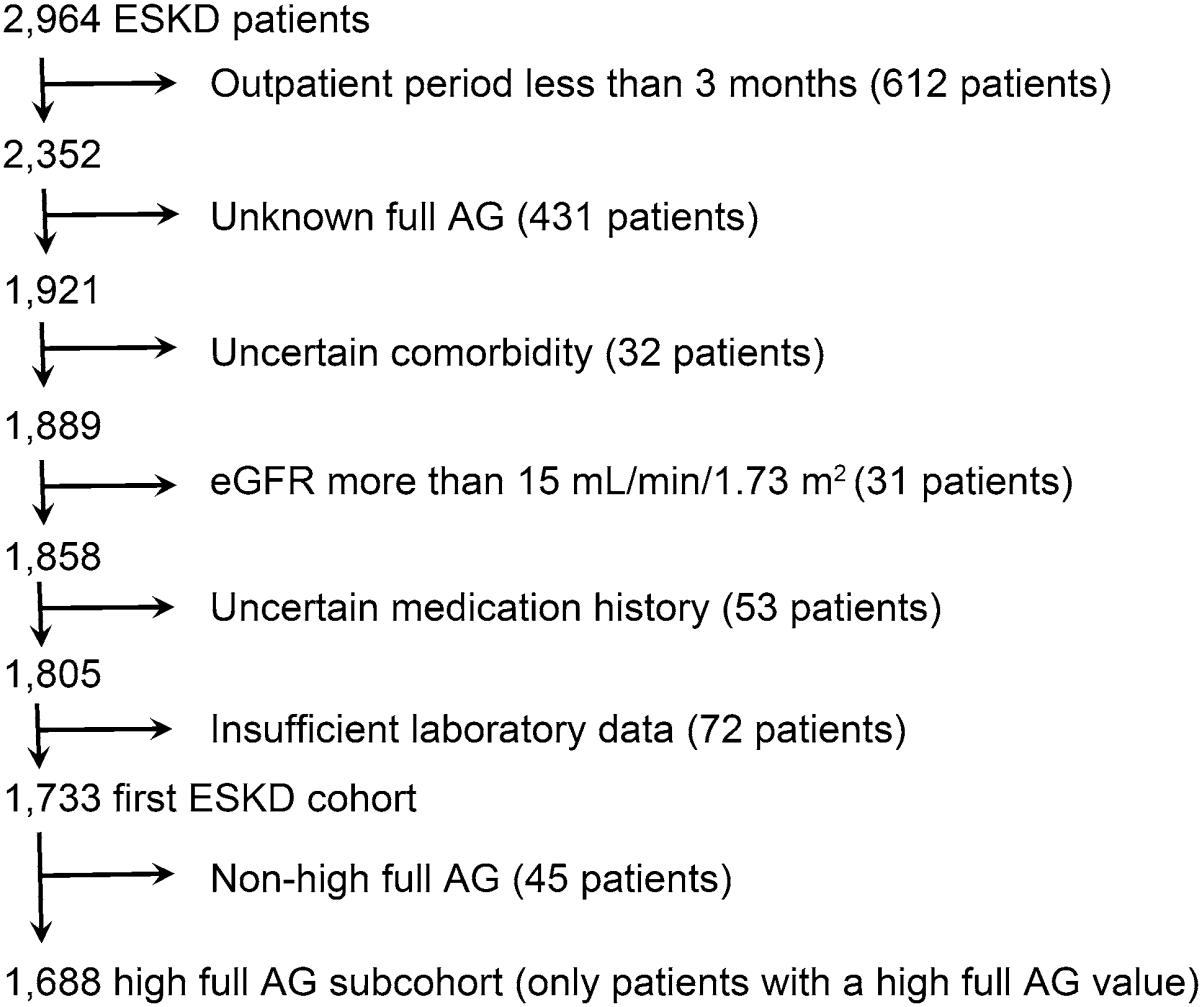
Flowchart showing the recruitment and screening of study participants. A total of 2964 non-dialysis patients with ESKD were recruited. Please note the difference between the first ESKD cohort and the second high full AG subcohort.

**Figure 2:**
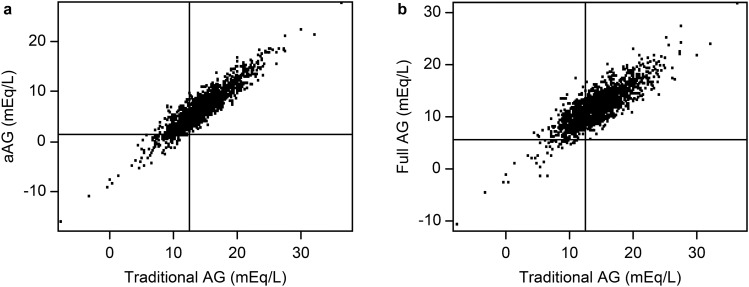
Diversity of the albumin-adjusted and full AG values against the traditional AG value in ESKD. (**a**) Traditional AG value against the aAG value. The added horizontal line indicates the upper limit of aAG (1.47 mEq/L). The added vertical line indicates the upper limit of traditional AG (12.42 mEq/L). The left upper graph area indicates a false-negative diagnosis by the traditional AG equation (aAG: albumin-adjusted AG). (**b**) Traditional AG value against the full AG value. The added horizontal line indicates the upper limit of full AG (5.69 mEq/L). The added vertical line indicates the upper limit of traditional AG (12.42 mEq/L). The left upper graph area indicates a false-negative diagnosis by the traditional AG equation.

First, we included 1733 patients with ESKD (Fig. 1), and their diverse albumin-adjusted and full AG values against traditional AG values in ESKD are shown in Fig. 2. ESKD patients showed a wide range of AG values.

Each categorized area distribution map was divided by the upper limit line of each AG value.

### High AG prevalence is altered by the AG equation

The patient number and the prevalence of high AG among the 1733 CKD stage 5 patients were 1177 (67.9%), 1596 (92.1%) and 1688 (97.4%) for the traditional > 12.42 mEq/L (Fig. 2a right area), albumin-adjusted > 1.47 mEq/L (Fig. 2a upper area) and full AG > 5.69 mEq/L (Fig. 2b upper area), respectively.

Among the non-high traditional AG patients (n = 556, 32.08% for traditional AG < 12.42 mEq/L), the patient number and the prevalence of high aAG > 1.47 mEq/L (Fig. 2a left upper area) and high full AG > 5.69 mEq/L (Fig. 2b left upper area) were 419 (75.4%) and 512 (90.85%), respectively. These patients received a false-negative diagnosis of a non-high AG state by the traditional AG equation.

### Performance of the traditional AG equation compared with that of the full AG and aAG equations

Even in ESKD patients who should develop high AG, the traditional AG equation often fails to detect a high AG state. Therefore, we examined its sensitivity, specificity and agreement in this ESKD cohort.

Table 1 shows the sensitivity, specificity, accuracy and Kappa coefficient of both traditional AG > 12.42 and aAG > 1.47, which were 0.70 vs 0.94, 0.98 vs 0.93, 0.7 vs 0.94, and 0.103 vs 0.44, respectively (full AG > 5.69 was used as a reference for the upper limit value). The Kappa coefficient value between traditional AG > 12.42 and full AG > 5.69 remained below 0.20, indicating poor reliability [Kappa 0.119 (95% CI 0.086–0.153)]. On the other hand, the Kappa value between aAG > 1.47 and full AG > 5.69 showed moderate interrater repeatability [Kappa 0.474 > 0.40 (95% CI 0.381–0.566)]. The Kappa value between traditional AG > 12.42 and aAG > 1.47 showed slightly strong repeatability [Kappa 0.330 < 0.4 (95% CI 0.284–0.376)]. These Kappa values demonstrated that the aAG or full AG equations are more accurate than the traditional AG equation for diagnosing the AG state in ESKD patients.

**Table 1:** Sensitivity, specificity, accuracy and agreement of high AG criteria between the full AG equation and the other 2 equations.

	Sensitivity	Specificity	Accuracy	Kappa	95% CI
High traditional AG	0.70	0.98	0.7	0.103	0.074–0.132
High aAG	0.94	0.93	0.94	0.440	0.350–0.529

*aAG* albumin-adjusted AG.

### Comparison of patient characteristics between the high full AG and non-high full AG groups

To examine the characteristics of patients with non-high full AG, 1733 ESKD first cohort of patients were divided into two groups by the full AG values: the non-high full AG (AG < 5.69, n = 45) group and the high full AG group (AG > 5.69, n = 1688). Their characteristics were compared (Table 2).

**Table 2:** Comparison of patient characteristics between the high full AG and non-high full AG groups.

	Overall	Non-high full AG group	High full AG	*p* value
Variables	(n = 1733)	< 5.69 mEq/L (n = 45)	> 5.69 mEq/L (n = 1688)	
Traditional AG (mEq/L)	14.3 ± 3.8	6.5 ± 4.0	14.5 ± 3.6	< 0.0001
aAG (mEq/L)	6.3 ± 3.9	2.6 ± 2.5	6.5 ± 3.6	< 0.0001
Full AG (mEq/L)	11.7 ± 3.5	2.9 ± 3.4	11.9 ± 3.1	< 0.0001
Male gender, n (%)	1214 (70.1)	34 (75.6)	1180 (69.9)	0.4141
Diabetes kidney disease, n (%)	741 (42.8)	26 (57.8)	715 (42.4)	0.0391
Outpatient period (years)	3.7 ± 3.8	2.8 ± 2.9	3.8 ± 3.8	0.1074
Age (years)	68. ± 13.1	63.0 ± 13.6	68.4 ± 13.1	0.0057
Systolic blood pressure (mmHg)	154.0 ± 24.0	154.1 ± 22.7	154.0 ± 24.1	0.9704
**Laboratory data**				
Leukocyte (10^3^/mL^3^)	6.35 ± 2.52	6.45 ± 3.54	6.34 ± 2.48	0.7710
Hemoglobin (g/dL)	9.02 ± 1.42	9.05 ± 1.37	9.02 ± 1.43	0.8679
C-reactive protein (mg/dL)	1.4 ± 3.3	0.72 ± 2.00	1.42 ± 3.35	0.1642
eGFR (mL/min/1.73 m^2^)	5.11 ± 1.86	5.39 ± 1.95	5.10 ± 1.86	0.2941
Uric acid (mg/dL)	8.43 ± 2.36	8.35 ± 2.12	8.43 ± 2.37	0.8317
Sodium (mEq/L)	138.1 ± 4.22	138.6 ± 3.4	138.1 ± 4.2	0.4120
Potassium (mEq/L)	4.57 ± 0.81	5.00 ± 0.87	4.56 ± 0.81	0.0003
Chloride (mEq/L)	104.7 ± 6.1	107.9 ± 4.7	104.6 ± 6.1	0.0005
Corrected calcium (mg/L)	8.55 ± 0.95	8.2 ± 0.94	8.56 ± 0.95	0.0132
Ionized calcium (mEq/L)	4.53 ± 0.53	4.20 ± 0.51	4.53 ± 0.53	< 0.0001
Phosphorus (mg/dL)	6.31 ± 1.79	6.44 ± 2.03	6.31 ± 1.79	0.6352
Phosphate (mg/dL)	3.67 ± 1.04	3.74 ± 1.18	3.67 ± 1.04	0.6352
Albumin (g/dL)	3.18 ± 0.58	3.65 ± 0.48	3.17 ± 0.58	< 0.0001
Bicarbonate (mEq/L)	19.1 ± 4.8	24.2 ± 4.7	19.0 ± 4.7	< 0.0001
**Medications**				
ESA, n (%)	1427 (82.3)	34 (75.6)	1393 (82.5)	0.2263
VDRA, n (%)	452 (26.1)	11 (24.4)	441 (26.1)	0.7999
CaCO_3_, n (%)	517 (29.8)	18 (40.0)	499 (29.6)	0.1309
Loop diuretics, n (%)	1191 (68.7)	32 (71.1)	1159 (68.7)	0.7264
Statin, n (%)	589 (34.0)	15 (33.3)	574 (34.0)	0.9252
Alkali, n (%)	682 (39.4)	16 (35.6)	666 (39.5)	0.5972
AST-120, n (%)	282 (16.3)	3 (6.7)	279 (16.5)	0.0497
CCB, n (%)	1344 (77.6)	33 (73.3)	1311 (77.7)	0.4918
ACEI, n (%)	205 (11.8)	3 (6.7)	202 (12.0)	0.2772
ARB, n (%)	1013 (58.5)	28 (62.2)	985 (58.4)	0.6032
RAAS inhibitors, n (%)	1092 (63.0)	30 (66.7)	1062 (62.9)	0.6069
RAAS inhibitors or loop or alkali, n (%)	1617 (93.3)	43 (95.6)	1574 (93.2)	0.5407

*aAG* albumin-adjusted AG.

Full AG value of each group were 2.9 ± 3.4 mEq/L for non-high full AG group vs 11.9 ± 3.1 mEq/L for high full AG group, respectively. Serum potassium, chloride, albumin, bicarbonate and frequency of DKD were significantly higher in the non-high full AG group. Interestingly, possible AG reducing medicine, RAAS inhibitors, Loop diuretics or Alkali were frequently prescribed in 1617/1733 patients (93.3%) and their prescribing rates were high in both high and non-high full AG group without significant difference (1574/1688, 93.2% vs 43/45, 95.6%, p = 0.5407).

On the other hand, age, serum corrected calcium, ionized calcium and AST-120 use were significantly lower in the non-high full AG group. There were no significant differences in gender, outcome patient period, systolic blood pressure, leukocyte count, hemoglobin, C-reactive protein, eGFR, uric acid, sodium, phosphate and the other medication use between two groups.

### Characteristics of patients with false-negative diagnosis of high AG

A total of 45 patients among first 1733 ESKD cohort were classified as having non-high full AG, so we excluded these 45 patients to make a complete high full AG subcohort comprised of high full AG patients alone (n = 1688) and explored how the traditional AG equation misdiagnoses the AG state in ESKD.

To examine the characteristics of misdiagnosed patients, all subcohort of patients with high full AG values were divided into two groups by the traditional AG values: the non-high AG (AG < 12.42, n = 512) group and the high AG group (AG > 12.42, n = 1176). Their characteristics were compared (Table 3).

**Table 3:** Comparison of patient characteristics between the high (traditional) AG and non-high (traditional) AG groups.

	Non-high AG	High AG	p value
Variables	< 12.42 (n = 512)	> 12.42(n = 1176)	
Traditional AG (mEq/L)	10.7 ± 1.4	16.1 ± 2.9	< 0.0001
aAG (mEq/L)	3.0 ± 1.7	8.0 ± 3.2	< 0.0001
Full AG (mEq/L)	9.3 ± 1.8	13.1 ± 2.9	< 0.0001
Male gender, n (%)	369 (72.1)	811 (69.0)	0.2007
Diabetes kidney disease, n (%)	237 (46.3)	478 (40.7)	0.0310
Outpatient period (years)	3.9 ± 4.3	3.7 ± 3.6	0.2338
Age (years)	70.4 ± 12.1	67.6 ± 13.4	< 0.0001
Systolic blood pressure (mmHg)	156.5 ± 24.7	152.9 ± 23.7	0.0050
**Laboratory data**			
Leukocyte (10^3^/mL^3^)	5.91 ± 2.04	6.53 ± 2.63	< 0.0001
Hemoglobin (g/dL)	8.95 ± 1.33	9.00 ± 1.51	0.2146
C-reactive protein (mg/dL)	1.0 ± 2.6	1.6 ± 3.6	0.0021
eGFR (mL/min/1.73 m^2^)	5.76 ± 1.86	4.81 ± 1.78	< 0.0001
Uric acid (mg/dL)	8.05 ± 2.03	8.59 ± 2.48	< 0.0001
Sodium (mEq/L)	138.3 ± 3.9	138.0 ± 4.4	0.2842
Potassium (mEq/L)	4.69 ± 0.76	4.50 ± 0.82	< 0.0001
Chloride (mEq/L)	106.8 ± 5.3	103.7 ± 6.2	< 0.0001
Corrected calcium (mg/L)	8.84 ± 0.76	8.43 ± 1.00	< 0.0001
Ionized calcium (mEq/L)	4.70 ± 0.46	4.45 ± 0.55	< 0.0001
Phosphorus (mg/dL)	5.39 ± 1.18	6.70 ± 1.86	< 0.0001
Phosphate (mg/dL)	3.14 ± 0.69	3.90 ± 1.08	< 0.0001
Albumin (g/dL)	3.07 ± 0.59	3.22 ± 0.57	0.0171
Bicarbonate (mEq/L)	20.7 ± 4.5	18.3 ± 4.6	< 0.0001
**Medications**			
ESA, n (%)	425 (83.0)	968 (82.3)	0.7297
VDRA, n (%)	134 (26.2)	307 (26.1)	0.9772
CaCO_3_, n (%)	146 (28.5)	353 (30.0)	0.5343
Loop diuretics, n (%)	352 (68.8)	807 (68.6)	0.9586
Statin, n (%)	182 (35.6)	392 (33.3)	0.3775
Alkali, n (%)	199 (38.9)	467 (39.7)	0.7444
AST-120, n (%)	80 (15.6)	199 (16.9)	0.5079
CCB, n (%)	409 (79.9)	902 (76.7)	0.1490
ACEI, n (%)	62 (12.1)	140 (11.9)	0.9052
ARB, n (%)	328 (64.1)	657 (55.9)	0.0017
RAAS inhibitors, n (%)	354 (69.1)	708 (60.2)	0.0005
RAAS inhibitors or loop or alkali, n (%)	481 (94.0)	1093 (92.9)	0.4503

*aAG* albumin-adjusted AG.

Age, systolic blood pressure, eGFR, potassium, chloride, corrected calcium, ionized calcium, bicarbonate, ARB use and CCB use were significantly higher in the non-high AG group. On the other hand, leukocytes, CRP, uric acid, albumin, phosphorus and phosphate were significantly lower in the non-high AG group. Possible AG reducing medicine, RAAS inhibitors or Loop diuretics or Alkali were frequently prescribed in this subcohort and their prescribing rates were high in both high and non-high traditional AG group with no significant difference (481/512, 94.0% vs 1093/1176, 92.9%, p = 0.4503).

Among the component molecules of the traditional AG equation, both serum chloride and bicarbonate were significantly higher and resulted in false-negative diagnoses of high AG in the non-high traditional AG group. Δchloride and Δbicarbonate between the non-high and high traditional AG groups were + 3.1 and + 2.4 mEq/L, respectively. As a result, these 2 molecules most powerfully influenced the decrease in traditional AG by 5.5 mEq/L in the non-high traditional AG group, which was almost equal to the differences in traditional AG values between the high and non-high traditional AG groups (5.4 mEq/L: 16.1 vs 10.7 mEq/L).

All of the other full AG components were higher in the non-high traditional AG group than in the high AG group (sodium + 0.3 mEq/L, potassium + 0.19 mEq/L, ionized calcium + 0.25 mEq/L, phosphate 0.76 mEq/L, albumin 0.15 g/dl × 2.5, total + 1.875 mEq/L contribution), and they contributed to an increase in the full AG value in the non-high traditional AG group from 10.7 to 12.575 mEq/L. Based on these full AG components and other factors, all patients in this subcohort diagnosed with non-high traditional AG values were rediagnosed with high full AG. Without these adjustments to the full AG equation, the traditional AG equation may often fail to screen the AG state precisely in CKD patients who frequently developed both hypoalbuminemia and electrolyte disorders, which influence the full AG value.

Univariable and multivariable logistic regression analyses in the 1688 high full AG subcohort showed several factors associated with a non-high traditional AG value in the non-high traditional AG group (Table 4). In brief, independent risk factors for a false-negative AG misdiagnosis were frequent ARB use, a relatively high eGFR, high chloride, high bicarbonate, high ionized calcium, high potassium, a relatively low blood leukocyte count, low serum albumin and low phosphate.

**Table 4:** Univariable and multivariable logistic regression models to identify the covariates associated with the non-high traditional AG value.

	Univariable model			Multivariable model		
	OR	95% CI	*p* value	OR	95% CI	*p* value
Age (years)	1.02	1.01–1.03	< 0.0001	1	0.99–1.01	0.714
C-reactive protein (mg/dL)	0.94	0.90–0.98	0.0009	0.99	0.93–1.04	0.6329
eGFR (mL/min/1.73 m^2^)	1.31	1.24–1.39	< 0.0001	1.15	1.06–1.25	0.0006
Uric acid (mg/dL)	0.9	0.86–0.95	< 0.0001	0.94	0.88–1.00	0.0551
Potassium (mEq/L)	1.35	1.19–1.54	< 0.0001	2.47	2.01–3.05	< 0.0001
Chloride (mEq/L)	1.1	1.08–1.13	< 0.0001	1.28	1.23–1.33	< 0.0001
Bicarbonate (mEq/L)	1.12	1.10–1.15	< 0.0001	1.38	1.32–1.45	< 0.0001
Albumin (g/dL)	0.64	0.54–0.77	< 0.0001	0.58	0.43–0.78	0.0004
Ionized calcium (mEq/L)	2.52	2.04–3.13	< 0.0001	1.92	1.38–2.71	0.0001
Phosphate (mg/dL)	0.38	0.33–0.44	< 0.0001	0.49	0.40–0.60	< 0.0001
Diabetic kidney disease	1.26	1.02–1.55	0.0313	1.04	0.78–1.40	0.7772
Systolic blood pressure (mmHg)	1.01	1.00–1.01	0.005	1	0.99–1.01	0.8193
ARB use	1.41	1.14–1.75	0.0016	1.38	1.05–1.81	0.0222
Leukocytes (10^3^/μL)	0.89	0.84–0.93	< 0.0001	0.88	0.82–0.94	0.0002

Low CRP, low uric acid, age, the presence of DKD and high systolic blood pressure were not independently associated with non-high AG in the multivariable logistic analysis, although they were significantly associated in the univariable logistic regression analysis.

### Associated factors with serum chloride in the high full AG subcohort

Next, we examined how these significant associated factors influence either the chloride or bicarbonate concentration separately, which mainly contribute to a decrease in traditional AG values and finally lead to false-negative diagnoses. Quantile analysis of serum chloride showed that frequency of high traditional AG, male gender, C-reactive protein, serum sodium, potassium, bicarbonate, ESA use, Loop diuretics use, Alkali use, ARB use and ACEI use were significantly associated with serum chloride concentration. Quantile analysis also showed that the frequency of high traditional AG decreased with increasing serum chloride concentration with mean frequency of high traditional AG (%) of 83.3, 76.0, 66.8, 53.5 for those in chloride quartiles ≤ 101, < 105, < 108, 108 ≤ mEq/L (p < 0.01), respectively.

These significant associated factors were used for univariable logistic regression analyses and resultant significant variates were used for further analysis. Multivariable logistic regression analyses showed that serum sodium, potassium and ARB use (beta coefficient 0.036, 95% CI 0.15–0.76, p = 0.0037) were independently associated with serum chloride positively (Table 5). On the other hand, bicarbonate (beta coefficient − 0.499, 95% CI − 0.68 to − 0.61, p < 0.0001) and C-reactive protein (beta coefficient − 0.075, 95% CI − 0.18 to − 0.09, p < 0.0001) were independently associated with serum chloride inversely. The other factors did not independently associated with serum chloride.

**Table 5:** Univariable and multivariable regression analysis of serum chloride.

Variable	Univariable model			Multivariable model		
	95% CI	*p* value	Beta coefficient	95% CI	*p* value	Beta coefficient
Male gender (%)	0.63 to 1.89	< 0.0001	− 0.095	− 0.01 to 0.65	0.0573	0.024
C-reactive protein (mg/dL)	− 0.31 to − 0.14	< 0.0001	− 0.124	− 0.18 to − 0.09	< 0.0001	− 0.075
Sodium (mEq/L)	0.86 to 0.97	< 0.0001	0.636	0.94 to 1.01	< 0.0001	0.675
Potassium (mEq/L)	2.07 to 2.75	< 0.0001	0.319	0.91 to 1.31	< 0.0001	0.147
Bicarbonate (mEq/L)	− 0.69 to − 0.58	< 0.0001	− 0.487	− 0.68 to − 0.61	< 0.0001	− 0.499
ESA (%)	0.33 to 1.86	0.005	0.068	− 0.13 to 0.67	0.1798	0.017
Loop diuretics (%)	− 1.93 to − 0.68	< 0.0001	− 0.099	− 0.62 to 0.03	0.0778	− 0.022
ARB (%)	0.40 to 1.57	0.0011	0.08	0.15 to 0.76	0.0037	0.036
ACEI (%)	0.06 to 1.85	0.037	0.051	− 0.24 to 0.69	0.3364	0.012
Alkali (%)	− 0.87 to 0.32	0.368	− 0.021			

### Associated factors with serum bicarbonate in the high full AG subcohort

Quantile analysis of serum bicarbonate showed male gender, Loop diuretics use, Alkali use, eGFR, serum chloride, potassium, ionized calcium and phosphate were significantly associated with serum bicarbonate concentration. ARB use did not associated with serum bicarbonate in this participants. Multivariable logistic regression analyses showed Alkali use (beta coefficient 0.077, 95% CI 0.39–1.09, p < 0.0001) and ionized calcium were independently associated with bicarbonate positively (Table 6). On the other hand, serum chloride (beta coefficient − 0.469, 95% CI − 0.39 to − 0.33, p < 0.0001), potassium and phosphate (beta coefficient − 0.342, 95% CI − 1.73 to − 1.36, p < 0.0001) were independently associated with serum bicarbonate inversely. Loop diuretics use was not independently associated with bicarbonate in the multivariable logistic analysis after adjusting serum chloride (beta coefficient 0.093, 95% CI 0.57 to − 1.31, p = 0.0826), although it was significantly associated in another model before adjusting serum chloride (beta coefficient 0.119, 95% CI 0.78–1.63, p < 0.0001).

**Table 6:** Univariable and multivariable regression analysis of serum bicarbonate.

Variable	Univariable model			Multivariable model		
	95% CI	*p* value	Beta coefficient	95% CI	*p* value	Beta coefficient
Male gender (%)	− 1.10 to − 0.12	0.014	− 0.060	− 0.47 to 0.30	0.6639	− 0.008
eGFR (mL/min/1.73 m^2^)	0.38 to 0.62	< 0.0001	0.197	− 0.02 to 0.20	0.1035	− 0.076
Chloride (mEq/L)	− 0.41 to − 0.34	< 0.0001	0.487	− 0.39 to − 0.33	< 0.0001	− 0.469
Potassium (mEq/L)	2.07 to 2.75	< 0.0001	0.328	− 0.76 to − 0.31	< 0.0001	− 0.092
Ionized calcium (mEq/L)	1.89 to 2.70	< 0.0001	0.262	0.66 to 1.32	< 0.0001	0.113
Phosphate (mg/dL)	− 1.83 to − 1.43	< 0.0001	− 0.36	− 1.73 to − 1.36	< 0.0001	− 0.342
Loop diuretics (%)	1.09 to 2.04	< 0.0001	0.155	0.57 to 1.31	0.0826	0.093
Alkali (%)	0.43 to 1.34	< 0.0001	0.092	0.39 to 1.09	< 0.0001	0.077

Though Alkali use independently associated with increase in serum bicarbonate in this analysis, univariable analysis of non-high traditional AG showed Alkali use had no significant association with non-high traditional AG value as described previously in this report.

The other factors did not independently associated with serum bicarbonate. Quantile analysis showed that the frequency of high traditional AG increased with increasing serum bicarbonate concentration with mean frequency of high traditional AG (%) of 56.5, 65.6, 73.4, 83.5 for those in bicarbonate quartiles ≤ 22, < 18.9, < 16, 16 ≤ mEq/L (p < 0.01), respectively.

### Associated factors with leukocyte count in the high full AG subcohort

Next, we examined how leukocyte count associate with other data. Quantile analysis of leukocyte count revealed that 3 AG value, frequency of diabetes kidney disease, outpatient period, age, systolic blood pressure, C-reactive protein, Uric acid, ionized calcium, phosphate, albumin, bicarbonate, ESA use, Alkali use, Loop diuretics use and AST-120 use were significantly associated with leukocyte count. Multivariable logistic regression analyses showed that frequency of diabetes kidney disease, ionized calcium and C-reactive protein were independently associated with leukocyte count positively. On the other hand, age, serum bicarbonate (beta coefficient − 0.061, t = − 2.08, 95% CI − 0.06 to − 0.01, p = 0.0113) and Alkali use were independently associated with leukocyte count inversely. The other factors did not independently associated with leukocyte count. As a result, leukocyte count inversely associated with either serum bicarbonate or Alkali prescription independently.

### Associated factors with ARB use in the high full AG subcohort

Next, we compared all parameters between ARB users and non-users, because ARB was the sole significant and independent associated medication with false negative diagnosis of high AG. As a result, ARB users showed significant lower AG values in all 3 equations and younger age. ARB users also showed significant increase in prevalence of DKD, statin use, CCB use, high potassium and high chloride. Multivariable logistic regression analysis showed prevalence of DKD, Statin use, CCB use, serum potassium and chloride were independently associated with ARB use positively. Age was independently associated with ARB use inversely. ARB users showed significant higher chloride, higher potassium, lower AG value and lower bicarbonate (without significance) than ARB non-users. These data support ARB could cause renal tubular acidosis type IV even in patients with ESKD who received frequent ARB prescription.

## Discussion

In this study, we have addressed both the frequency and possible mechanism of false-negative diagnosis of high AG in non-dialysis patients with ESKD. These ESKD patients with an eGFR less than 15 mL/min/1.73 m^2^ showed a high prevalence (1688/1733: 97.4%) of high full AG values just before initiating dialysis. However, traditional AG could detect high AG in only approximately 67.9% of these patients. Indeed, the specificity of traditional AG was high, but the sensitivity, accuracy and Kappa coefficient were low. There were large differences in the sensitivity, accuracy and Kappa coefficient values used to diagnose high AG between traditional AG and aAG. Xu et al*.*^11^ also reported that the sensitivity of traditional AG > 12 mmol/L to screen for lactic acidosis was low (61%), similar to our results. Our hypothesis was that the traditional AG equation often diagnose AG state false-negatively in patients with ESKD. Consistent with our hypothesis, the Kappa coefficient value between traditional AG and full AG remained below 0.20, indicating poor reliability. The Kappa value demonstrated that the albumin-adjusted or full AG equations were more accurate in diagnosing the AG state than the traditional AG equation in our patients with ESKD.

The reference range of AG values was an important factor in this study, and the range of traditional AG has been reported to vary, with the upper limit ranging from 10 to 20^2,12–16^. We referred to the data of Abramowitz et al*.*^7^ as the reference range because they showed detailed data concerning the reference range of AG and calculated all 3 kinds of AG means and standard errors in accordance with a large number of participants (n = 5288) with an eGFR of 90–119 mL/min/1.73 m^2^. To our knowledge, there have been no other reports that show the reference ranges of both full AG and aAG in these participant number scales.

If the upper limit was set to 10 to obtain the maximum sensitivity, specificity, accuracy and Kappa value, each value was 0.91, 0.84, 0.91, and 0.328, respectively. However, this Kappa value could not reach that of aAG, indicating that the traditional AG equation could not overcome the aAG equation at the point of agreement in patients with ESKD. To obtain higher agreement, the upper limit of traditional needs to be set to under 10 mEq/L, an unreported range to date. Previously, Adams et al*.*^17^ reported that using revised AG > 6 mEq/L is sensitive but not specific to lactic acidosis. These reference ranges of traditional AG settings lose their specificity against full AG, as reported by Adams et al*.*^17^*,* and these settings accounted for the outside of the 95% of values from a normal population. The reference range of traditional AG reported to date never gains more agreement beyond aAG.

We draw ROC curve and calculated both traditional AG cutoff value and albumin-adjusted AG cutoff value for full AG > 5.69 mEq/L using our patients with ESKD and resultant each cutoff values were 11.0 and 1.8 mEq/L, respectively. Using these cutoff value, we re-evaluated the sensitivity, specificity, accuracy and agreement of high AG criteria. Both the patient number and the prevalence of high AG among the 1733 CKD stage 5 patients were 1419 (81.9%), 1565 (90.3%) and 1688 (97.4%) for the traditional > 11 mEq/L, albumin-adjusted > 1.8 mEq/L and full AG > 5.69 mEq/L, respectively. Thus, the traditional AG equation could not overcome either the albumin-adjusted or full AG equation to accurately diagnose the AG state in ESKD.

Among the non-high traditional AG patients (n = 312, 18.5% for traditional AG < 11 mEq/L), the patient number and the prevalence of high aAG > 1.8 mEq/L and high full AG > 5.69 mEq/L were 157 (50.3%) and 269 (86.2%), respectively. These results also re-confirmed many non-dialysis patients with ESKD received a false-negative diagnosis of high AG by the traditional AG equation.

Next, we have addressed possible mechanism of false-negative diagnosis of high AG in non-dialysis patients with ESKD. First, possible AG reducing medicine, RAAS inhibitors or Loop diuretics or Alkali are frequently prescribed in patients with ESKD in the world. In fact, these prescription rate in our participants was very high in both high and non-high traditional AG group with no significant difference (481/512, 94.0% vs 1093/1176, 92.9%, p = 0.4503).

Independent factors associated with a false-negative AG misdiagnosis were frequent ARB use, a relatively high eGFR, high serum chloride, high bicarbonate, high ionized calcium, high potassium, a relatively low blood leukocyte count, low serum albumin and low phosphate. Table 3 showed mean Δchloride (+ 3.1 mEq/L) and mean Δbicarbonate (+ 2.4 mEq/L) between the non-high and high traditional AG groups resulted in a decrease in traditional AG by total 5.5 mEq/L in the non-high traditional AG group, indicating the main key contributors to the low traditional AG value were high chloride and high bicarbonate among these factors. The other associated factors showed smaller differences than these 2 key molecules. Therefore, we examined how these 2 key molecules increased in the next step.

Multivariable logistic regression analyses of serum chloride revealed ARB use, high sodium, high potassium, low bicarbonate and low C-reactive protein may cause false-negative diagnosis of high AG via elevation in serum chloride. Moreover, Loop diuretics use, Alkali use, high ionized calcium, low chloride, low potassium and low phosphate may cause false-negative diagnosis of high AG via elevation in bicarbonate. Potassium chelate use, phosphate binder use, calcium use also may cause non-high AG via elevation in bicarbonate, although we do not have these data.

Recently, ARBs are used in Japanese patients with CKD more frequently than ACEIs because ACEI-induced cough is frequently observed in East Asians^6,18^. In our full AG subcohort, ARBs were prescribed more frequently than ACEIs. ARBs are known to cause type IV renal tubular acidosis (RTA)^19^, so it is reasonable to hypothesize that the frequent use of ARBs might cause relatively high potassium, relatively high chloride and non-high AG in the non-high AG group even in patients with ESKD. A higher prevalence of DKD in the non-high AG group may be associated with both frequent ARB use and a relatively low range of serum albumin due to the frequent development of nephrotic syndrome.

Recently, as ARB prescribed more frequently, both traditional AG value and frequency of false-negative diagnosis of high AG in patients with CKD may increase.

Multivariable logistic regression analyses of leukocyte count indicated that leukocyte count independently associated with both serum bicarbonate and Alkali use, suggesting inflammatory disease could induce high AG state via metabolic acidosis, that is, non-dialysis patients with ESKD may have non-high AG value if they complicated no inflammatory disease.

Inverse association between leukocyte count and Alkali use suggests nephrologists might stopped sodium bicarbonate prescription for patients complicated with any inflammation who are easy to develop congestive heart failure.

Farwell and Taylor^20^ reported that high AGs and a low bicarbonate level were associated with both a high leukocyte count and a high CRP level in healthy individuals. For example, serum lactate is a well-known anion which increase according to the grade of inflammation and AG value^21^. In this study, we also showed that the traditional AG value was positively associated with both the leukocyte count and CRP level in patients with ESKD. The association between high AG, high CRP and a high leukocyte count and a low eGFR detected in this study may also support the increased risk of mortality by high AG, as previously reported^7,8^.

In conclusion, we demonstrated that the traditional AG equation frequently misdiagnoses a high AG state in non-dialysis patients with ESKD. More than half a century ago, traditional AG equation was formulated before the development of RAAS inhibitors^22^. Today’s patients with ESKD had various complications and received various treatments that might influence on traditional AG value. Clinicians should pay attention to any of medicines, incidental inflammatory findings and eGFR which may influence on AG level, if they calculate AG value of the non-dialysis patients with ESKD. We recommend using a more accurate AG equation if ESKD patients present non-high traditional AG values.

### Limitations

This study suggests that ARB use, the eGFR and the leukocyte count contribute to high chloride and bicarbonate levels, but this observational cross-sectional study could not precisely demonstrate the cause of the false-negative diagnosis of high AG. This ESKD cohort comprised a small population with non-high full AG even just prior to initiating dialysis, but we could not diagnose each cause of the non-high traditional AG value.

We identified several candidate factors associated with low AG levels and a misdiagnosed AG state in ESKD, but we did not measure all of the serum organic anion concentrations involving uremic toxins, so we could not precisely show what unmeasurable anions were increased in our ESKD cohort.

We did not have information on renin inhibitors and antialdosterone inhibitors. And the small population of ACEI users (12%) in this cohort might have influenced the results.

We did not have proper control participants with reference range of AG without ESKD to calculate cutoff value of each AG equation. The reference ranges for the three AG equations were determined by using 5288 samples with an eGFR 90–119 mL/min/1.73 m^2^ described by Abramowitz et al*.*^7^ as the mean ± 2 SE.

Despite these limitations, our study demonstrated that traditional AG equation often diagnose AG state false-negatively in patients with ESKD. Today’s frequent use of AG-reducing medicines may conceal high AG state in non-dialysis patients with ESKD.

## Methods

### Database

This study used a cross-sectional, observational, multicenter design. Clinical information and hematological data were collected at the institutional level immediately before the first hemodialysis session according to the JSTART database (JMP 9.0, SAS Institute Inc., Cary, NC, USA). Each patient’s information was labeled with only the institution and patient number to protect the patient’s privacy. We updated the JSTART database by adding new data. In this study, we included 2964 Japanese patients between January 1, 2009 and September 30, 2017. This study was performed in accordance with the Declaration of Helsinki. The Ethics Committee for Clinical Research of Jikei University School of Medicine approved this study [permission no. 25-343 (7849)]. The requirement to obtain informed consent from the patients was waived because of the retrospective nature of the study. Instead, all individual participants were provided the opportunity to opt out of this study.

A total of 2964 non-dialysis patients with ESKD were recruited. The following inclusion criteria were selected: (1) Japanese patients more than 20 years old and (2) patient records for the following data: data needed to calculate full AG, medications, age, sex, presence of diabetic kidney disease (DKD), duration of nephrologist follow-up, systolic blood pressure, and laboratory data [leukocyte count, hemoglobin, albumin, urea nitrogen, creatinine, sodium, potassium, chloride, corrected calcium, phosphorus, bicarbonate and C-reactive protein (CRP)]. The estimated glomerular filtration rate (eGFR) was calculated using the new Japanese equation^23^: eGFR (mL/min/1.73 m^2^) = 194 × Cr^−1.094^ × age^−0.287^ (× 0.739 for women).

As a result, 1231 patients were excluded from the analysis due to having an outpatient period less than 3 months (612 patients), having no data to calculate full AG (431 patients), having an uncertain comorbidity (32 patients), having an eGFR more than 15 mL/min/1.73 m^2^ (31 patients), lacking data regarding medication use (53 patients), and lacking laboratory data (72 patients). Therefore, of the 2964 patients evaluated, 1733 Japanese patients satisfied the inclusion criteria and were included in the analysis (Fig. 1).

### Three different AG equations and their reference ranges

The AG was calculated as follows^1,7^:

$$\begin{gathered} {\text{traditional AG}} = {\text{serum sodium }}\left( {{\text{mEq}}/{\text{L}}} \right) - \left( {{\text{serum chloride }}\left( {{\text{mEq}}/{\text{L}}} \right) + {\text{serum bicarbonate }}\left( {{\text{mEq}}/{\text{L}}} \right)} \right);{\text{ aAG}} = {\text{traditional AG}} - \left( {{2}.{5} \times {\text{serum albumin }}\left( {{\text{g}}/{\text{dL}}} \right)} \right); \hfill \\ {\text{and full AG}} = {\text{aAG }} + {\text{ serum potassium }}\left( {{\text{mEq}}/{\text{L}}} \right) + {\text{ionized calcium }}\left( {{\text{mEq}}/{\text{L}}} \right) - {\text{serum phosphate }}\left( {{\text{mEq}}/{\text{L}}} \right); \hfill \\ {\text{ionized calcium }}\left( {{\text{mEq}}/{\text{L}}} \right) = \left[ {0.{5} \times \left( {{\text{total calcium }}\left( {{\text{mg}}/{\text{dL}}} \right) + 0.{8} \times \left( {{4} - {\text{serum albumin }}\left( {{\text{g}}/{\text{dL}}} \right)} \right)} \right]/{2}} \right); \hfill \\ {\text{and serum phosphate }}\left( {{\text{mEq}}/{\text{L}}} \right) = \left( {0.{323} \times {\text{serum phosphorus }}\left( {{\text{mg}}/{\text{dL}}} \right)} \right) \times {1}.{8}. \hfill \\ \end{gathered}$$

The reference ranges for the three AG equations were determined by using 5288 samples with an eGFR 90–119 mL/min/1.73 m^2^ described by Abramowitz et al*.*^7^ as the mean ± 2 SE. The values were 12.08 (11.74–12.42), 1.13 (0.77–1.47), and 5.35 (5.01–5.69) mEq/L for the traditional, albumin-adjusted, and full AG equations, respectively. We used these upper limits to distinguish high AG criteria in this study. Then, the patient number and the prevalence of high AG among a total of 1733 ESKD patients were calculated for the traditional > 12.42 mEq/L, albumin-adjusted > 1.47 mEq/L and full AG > 5.69 mEq/L equations, respectively.

### Creating a secondary high full AG subcohort

Next, we created a secondary subcohort comprised of only patients with high full AG values to explore factors associated with misdiagnosis of a traditional AG state. Forty-five patients with non-high full AG were excluded from the ESKD cohort, and only 1688 patients with high full AG were selected as the high full AG subcohort. In this subcohort, 512 patients received a false-negative diagnosis and were classified as the non-high traditional AG group. This false-negative non-high traditional AG group was compared to the high traditional AG group, and the resultant significant explanatory variables associated with a false-negative diagnosis as non-high AG were selected and subjected to logistic regression analysis.

Quantile analyses of serum chloride, bicarbonate, eGFR and leukocyte count were performed and we compared all parameters between ARB users and non-users. Resultant significant associated factors were used for further univariable and multivariable logistic regression analyses.

### Variables

The following explanatory variables were evaluated: sex; age; comorbid DKD; nephrologist follow-up period; systolic blood pressure; eGFR; serum sodium; potassium; chloride; bicarbonate; ionized calcium; serum phosphate; albumin: CRP; uric acid; and hemoglobin concentration.

The following information regarding the recent medications ordinarily used for CKD patients was also collected: erythropoiesis-stimulating agents (ESAs), renin–angiotensin–aldosterone system (RAAS) inhibitors [angiotensin-converting enzyme inhibitors (ACEIs) or angiotensin II receptor blockers (ARBs)], calcium channel blockers (CCBs), loop diuretics, other antihypertensive agents (alpha blockers and/or beta blockers and/or other hypertensive drugs), vitamin D receptor activators (VDRAs), calcium carbonate (CaCO_3_), and AST-120. AST-120 (Daiichi-Sankyo Industry Co., Tokyo, Japan) is a carbonaceous adsorbent that is used to treat patients with CKD. We also setup a group who prescribed any of RAAS inhibitors or Loop diuretics or Alkali which may reduce AG value, and examined their prescription rate.

Explanatory factors associated with non-high AG values on the univariable analysis were subsequently included in a logistic regression model. Beta coefficients or odds ratios (ORs) and 95% confidence intervals (CIs) were determined using univariable and multivariable logistic regression models to identify the covariates that were associated with the non-high traditional AG value.

### Statistics

Statistical analyses were performed using JMP 9. Data are expressed as the means ± standard deviations or numbers (percentages) of patients. Comparisons across the various groups were performed using the Pearson chi-square test for categorical data and the Dunnett test for continuous data. Comparisons across the tertile analysis were performed using the Cochran–Armitage trend test for categorical data and the Dunnett test for continuous data. All tests were two-tailed, and a p value < 0.05 was considered significant. Factors that were associated with non-high AG values on the univariable analysis were subsequently included in a logistic regression model. When testing interrater repeatability, the percent agreement and Cohen’s Kappa value were calculated. The sensitivity, specificity, accuracy and Kappa coefficient of both traditional AG > 12.42 mEq/L and aAG > 1.47 mEq/L were calculated, and full AG > 5.69 mEq/L was used as a reference for the upper limit value.

## Data Availability

The datasets generated and analyzed during the current study are not publicly available. The datasets are available from the corresponding author on reasonable request when the aim is to verify the published results.
